# Can’t We Do Better? A cost-benefit analysis of proposal writing in a competitive funding environment

**DOI:** 10.1371/journal.pone.0282320

**Published:** 2023-04-19

**Authors:** Gerald Schweiger

**Affiliations:** Graz University of Technology, Graz, Austria; Consejo Superior de Investigaciones Cientificas (CSIC), SPAIN

## Abstract

This article presents an analysis of third-party funding in Austria for energy research, including an analysis of the costs and benefits of writing proposals and of the trust proposal applicants place in the application process. For this purpose, applicants from research and industry applying for government-funded energy research grants in Austria were surveyed. Preparing a new proposal takes about 50 working days; at the current success rate, about 300 person-days are spent preparing proposals for a single proposal to be funded.More than 90% of researchers perceive that they currently spend too much time preparing proposals and only 10% of researchers believe that the current competitive third-party funding system has a positive effect on the quality of research. Furthermore, researchers have limited trust in the objectivity of proposal review processes.

## 1 Introduction

The transition towards a sustainable energy system is a key challenge of the 21st century and the European Union intends to become a global leader on the path to sustainable energy systems. The motivation for this article is based on the observations of two growing trends: (1) massive funding of energy-related research and development in many countries, and (2) a change in funding mechanisms, leading to increased competitive third-party funding.

The European Green Deal is providing 1 billion euros to mobilise the European research and innovation community [1]. In 2021, the Austrian government spent almost half of the budget for applied and industrial research (a total of 738 million) on climate-related projects, with the largest share going to projects in the fields of energy and the environment [2]. In 2020, the German government spent around 1.2 billion euros on research, development and demonstration of energy technologies [3].

In recent decades, the funding mechanisms of universities across Europe have changed, with the goal of improved performance, with the assumption that a competitive environment (i) leads to better outcomes if the best performer is funded, and (ii) that this incentivizes others to perform better [4]. An important aspect of this competitive research environment is third-party funding from public and private sources [5].

This article presents an analysis of third-party funding in Austria for energy research. Austria has three technical universities; as of 2020, the share of third-party funded employees in these three universities is 33%. Several concerns are addressed in the literature. (a) Research shows that competitive research does not always result in a higher level of efficiency and productivity [5]. A recent study shows that increasing the share of competitive funding instead of institutional funding does not result in a more efficient system [6]. (b) Funding systems appear to largely fail in their task of reliably ranking the relative research quality of proposals. In their well-known experimental study, Cole et al. showed that “[…] getting a research grant depends to a significant extent on chance” [7]. Other studies came to similar conclusions [7–10]. In the literature on grant funding, ‘chance’ is not defined in a mathematical manner where chance is the occurrence of events in the absence of any obvious intention or cause. The literature on grant funding refers to chance from the applicant’s point of view: even if the reasons for grant decisions could be theoretically explained (e.g., preferences and biases of experts), the decisions from the applicant’s point of view appear random. A study in health and medicine research found that allocating funding is costly and somewhat random [8]. A study on grant funding from the National Institutes of Health in the US found no agreement among reviewers in evaluating the same application [11]. (c) An increased competitive funding environment could cause universities to adopt a mainstream strategy, following trends and buzzwords to attract students and third-party funding [12].

### 1.1 Main contribution

The Austrian Research Promotion Agency, or Forschungsfördergesellschaft (FFG), is the central organization for promoting and financing research, development, and innovation in the field of applied and industrial research in Austria; FFG is 100% owned by the Republic of Austria. The following study investigates competitive third-party funding in Austria in the field of energy research.For this purpose, the author surveyed the Austrian research community and industry on topics such as the costs and benefits of writing proposals and the confidence applicants have in the proposal review process. In addition, research calls in the field of energy research and the general third-party funding system in Austria were analyzed. This paper thus contributes to the broader discussion about different funding mechanisms, and specifically whether certain funding mechanisms outweigh their cost on a societal and individual level [6,13–15]. Furthermore, the paper contributes to the body of literature on analyzing the process of grant proposals [8,16–18].

### 1.2 Related work

Ted von Hippel and Courtney von Hippel [17] surveyed psychologists and astronomers active in applying for federally funded research; for better comparability, the results given in hours in the study were converted into days (1 day = 8 hours).They found that the average proposal takes 15 Principal Investigator days and 7 Chief Investigator days to write; the time spent writing was not related to whether the grant was funded. Effort did translate into success, however, as academics who wrote more grants received more funding. Herbert et al. [16] analyzed research funds awarded by the National Health and Medical Research Council. Their survey shows that preparing a new proposal took an average of 38 days while a resubmitted proposal took 28 days. Their results confirm the findings of previous studies [8,18]. The funding system in Austria is of central importance for this study. Wiener et al. [5] analyzed the funding structure of Austrian universities and how third-party funding has changed over time. Their results show that the overall level of third-party funding has increased between 2007 and 2017.

## 2 Method

The study focused on research proposals submitted to energy related research calls by different actors to the Austrian Research Promotion Agency FFG. Two categories are defined: research and industry. The research category includes universities, applied universities (Fachhochschulen), non-university research institutes, and research companies. A research company is defined as a company where more than 50% of the employees work on funded research projects or projects funded by public authorities and governmental bodies. These companies were explicitly contacted by email to confirm their status; if no confirmation was received, they were listed as regular companies in the industry category.

### 2.1 Austria’s funding programs in the field of energy research

Firstly, (a) relevant calls in the field of energy research were identified and (b) available data regarding projects, funding, funding rates, acceptance rates, etc. were analyzed. For this purpose, various websites and databases were analyzed [19–22].

### 2.2 Empirical survey

**Fig 1:**
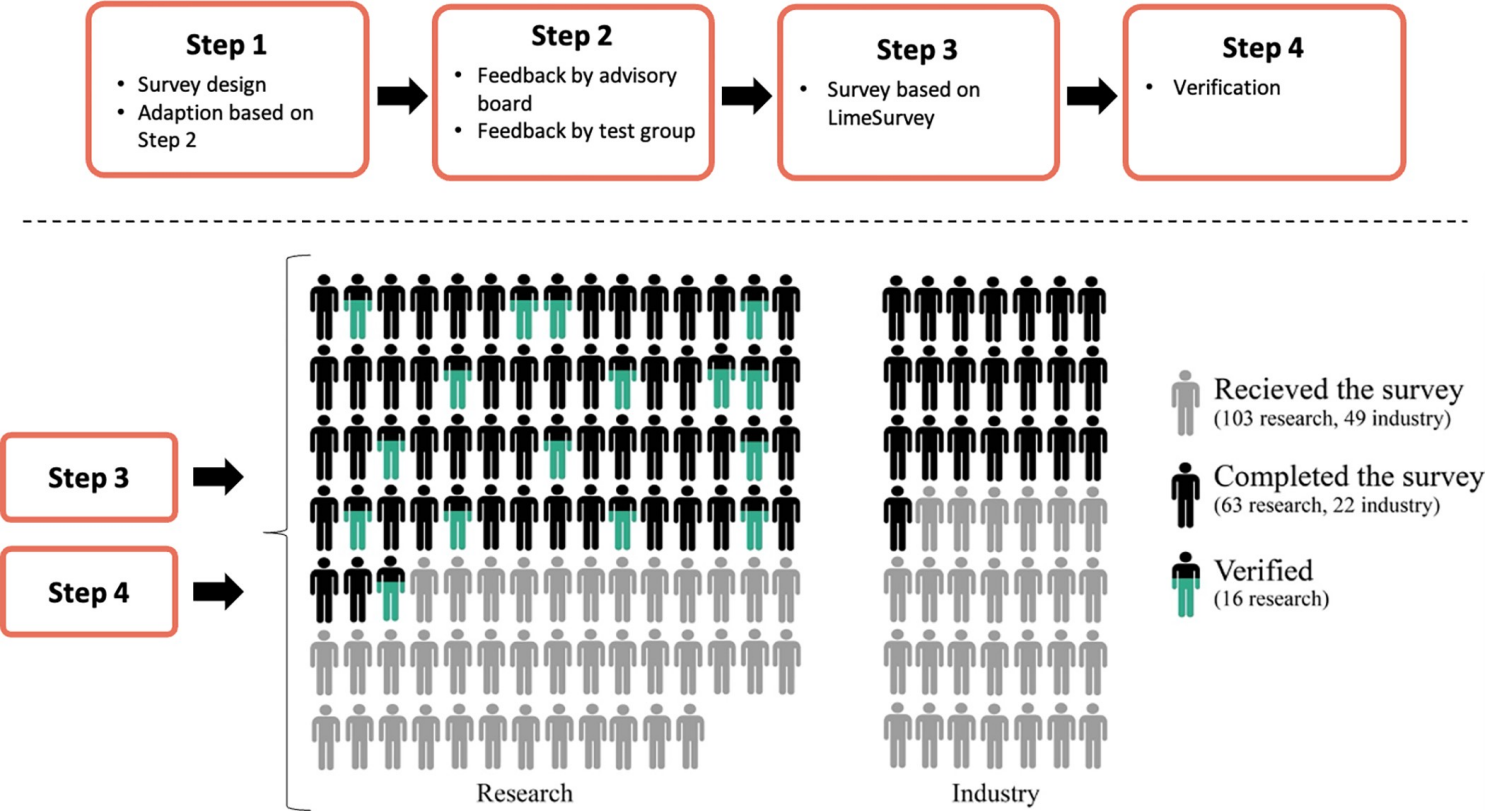
Four-step process of the empirical survey.

The research design followed a four-step process and is shown in Fig 1. The survey was in German. Raw data and a translation of the survey into English are available at https://github.com/GersHub/Third-Party-Funding.

Survey questions were formulated either as 5-point Likert scale questions (strongly disagree, …, strongly agree), as single/multiple choice questions, or as questions requiring numerical responses (Step 1).

All survey questions were (i) reviewed by an advisory board consisting of two experts from social science and one from applied energy research and (ii) were tested on a test group consisting of six people: two experts from social science and four from applied energy research who submit research proposals several times a year. The questions were then adapted based on feedback from the test group.

In Step 3, suitable participants in the field of energy research were selected based on two research project databases [19,20]. Potential participants were either mentioned directly in the project description as the contact person of the respective organization or they were listed on the website of the organization as project leader. In total, 103 people from the category “research” and 49 people from the category “industry” received the link to an online survey constructed with the survey tool LimeSurvey. After receiving a final reminder by email, 63 people from research and 22 from industry completed the survey, leading to a response rate of 66% for research and 45% for industry.

In Step 4, responses from researchers on the question about how much time participants and their organization spend on preparing proposals showed some outliers–the method to detect outliers is details in Section 2.2.3. It is important to note that the survey for industry was sent after the survey for researchers. Here, a kind of "unit" was additionally defined for the question ("answer in days"); answers from industry do not show any possible outliers. The hypothesis of the author was that the participants answered the question in hours and not in days, as the question had explicitly stated (original questions are listed online: https://github.com/GersHub/Third-Party-Funding). The goal was to test the hypothesis in approximately 20% of the original respondents. As the survey was anonymous, non-responders could not be identified in advance. Therefore, 30 out of the 103 people were randomly selected (using Python’s random sample module) and contacted. These participants were asked if it was to them that the answer was in days and not hours. Furthermore, participants were asked to answer the following question again by mail: “How many days (1 day = 8 hours) did your organization spend, in total (including your time), preparing proposals (as lead and/or partner)”.

#### 2.2.1 Measures research

At the beginning of the survey, participants from research were asked whether they had been involved in an FFG project application in the field of energy in the last 3 years as (a) project lead, (b) project partner, or (c) both, project lead and project partner. Based on this response, the questions were automatically adjusted; if the person was not involved in any project, the survey was terminated. To protect anonymity, no names or personal details were included in the survey. Further questions were related to the following considerations:

- **Demographics**: Organization, current position, number of projects they submitted during the last 12 months, etc.
- **Project details:** The survey asked participants for all project specific questions to refer to their most recent FFG project application in the field of energy (regardless of whether the project was approved, rejected, or still under evaluation) in which their organization was the lead partner/project partner. The question did not refer to a specific funding program, as the application process for funding programs in the field of energy is very similar. The survey asked for participations details about the project such as: How many partners participated in the project? How big was the budget for their organization? To which funding program did they apply? Was it a resubmission or a new proposal?
- **Time expenditure**: The survey asked participants to provide an estimate of the total number of days they and their organization spent in total on preparing proposals; similar to [16] it was explicitly mentioned that this includes everything related to writing a proposal such as background reading, data analyses, writing the proposal, preparing the budget, etc.
- **Miscellaneous:** In addition, various questions were asked that could not be clearly assigned to a specific category. These included questions about the non-financial benefits of proposal writing, or the proposal review process.

#### 2.2.2 Measures industry

In contrast to the survey for research, participants from industry were asked only about their roles as project partners and not as project leads, because participants from industry generally dedicate less time to survey work [23]. In order to increase the response rate among participants from industry the survey was shortened compared to the research survey, as a high rate of non-responses increases the probability of statistical biases [24]. The questions on demographics, project details, and time expenditure were the same as the research survey. Questions about the time required for proposal preparation were asked in the survey for researchers read as follows: " How many days (1 day = 8 hours) did […]”. The questions in the *Miscellaneous* category were different. The focus here was on the role of research for the respective organizations and their various motivations for research projects.

#### 2.2.3 Outlier detection and survey data cleaning

Only the responses to the question on how much time people spent on preparing proposals showed possible outliers. Depending on the application and the number of observations in the data set, there are different approaches to detect outliers [25]. In this work, two outlier detection methods were applied: (a) A response is defined as a mild outlier (based on the definition in [26]) if it is outside the interval (*Q*_1_−1.5×*IQR*, *Q*_3_+1.5×*IQR*); Q_1_ is the lower quartile; Q_3_ is the upper quartile; the Interquartile Range (IQR) is the difference between Q_3_ and Q_1_. (b) A response is defined as an extreme outlier (based on the definition in [26]) if it is outside the interval (*Q*_1_−3×*IQR*, *Q*_3_+3×*IQR*). Next, the respective medians of the remaining data were compared. In a final step, the minimum, maximum and median values were compared with the data of the verification responses (see step 4 in Fig 1).

In this work, survey data cleaning consists of identifying and removing responses from participants who did not answer the questions thoughtfully. Therefore, (a) data of participants who showed straightlining [27] were removed. Straightlining is defined as providing the same answer to every question using the same response scale [28]. If respondents showed straightlining, all responses were removed from the analysis, not just the questions that showed straightlining. (b) Responses from participants who most likely answered a question incorrectly because they answered in hours instead of days were removed. (c) Contradictory responses were removed; this was the case for responses whose answer to the question, “How many days (1 day = 8 hours) did you (just you, not your institution’s team) spend working on the proposal?” was greater than the answer to the question “How many days (1 day = 8 hours) did your organization spend, in total (including your time), working on the proposals?”.

#### 2.2.4 Threats to validity

At each step of the empirical survey, potential threats to validity are considered and mitigated where possible. A critical step in a empirical survey is the selection of participants. In this study, potential participants were either named directly in the project description as the contact person for the respective organization or they were listed on the organization’s website as the project leader. Since all funded projects in the respective research programs were evaluated through the databases of the funding organizations, there is no bias toward certain projects having higher public visibility.

There is no guaranteed way to avoid nonresponse bias; however, a high response rate minimizes the bias. The survey had a response rate of 66% for research and 45% for industry, which is considered high compared to similar studies. Similar studies analyzing grant writing costs and benefits do not contact project leaders directly but, rather, through association and interest groups. In some studies, the response rate could not be determined due to a lack of information on the number of people contacted [17]. Another study contacted offices of universities and research institutes [16], resulting in a response rate of only 16% of the submitted proposals.

It is critical to identify outliers to avoid bias in the data. In this work, the quantification of the time needed for writing proposals is of crucial importance. Therefore, besides a two-step method for the detection of outliers, an additional step for verification was included (see Section 2.2.3) to increase confidence in the results.

There is not enough data in this study to draw a general conclusion regarding whether there are differences in how long it takes researchers to prepare new proposals compared to resubmissions. Results from previous studies in medical research show that resubmission takes 25% less time than the submission of a new proposal [16]. Compared to basic research proposals (e.g., in physics or medicine), where projects are usually adjusted after rejection based on reviewer comments, applied research proposals additionally require significant content adjustments, as the focus of the research call usually changes significantly from call to call. That being said, the author is cognizant that this is a working hypothesis, not yet evidence-based.

## 3 Results and discussion

Chapter 3.1 presents the analysis of Austria’s funding programs in the field of energy research and chapter 3.2 presents the results of the survey.

### 3.1 Austria’s funding programs in the field of energy research

The following research calls in the field of energy were identified and analyzed below: “Stadt der Zukunft (Eng.: city of the future)”, “Energieforschung (Eng.: energy research)”, and “Vorzeigeregion (Eng.: flagship region). Responses from the survey (see Section 3.2) also indicate that these are the most important research challenges in the field of energy research. However, the aim of this analysis is not to provide a complete overview of calls in which projects in the field of energy can be submitted, but rather to provide an overview of project compositions (research institutes, industries, etc.), project budgets, acceptance rates, etc.

**Fig 2:**
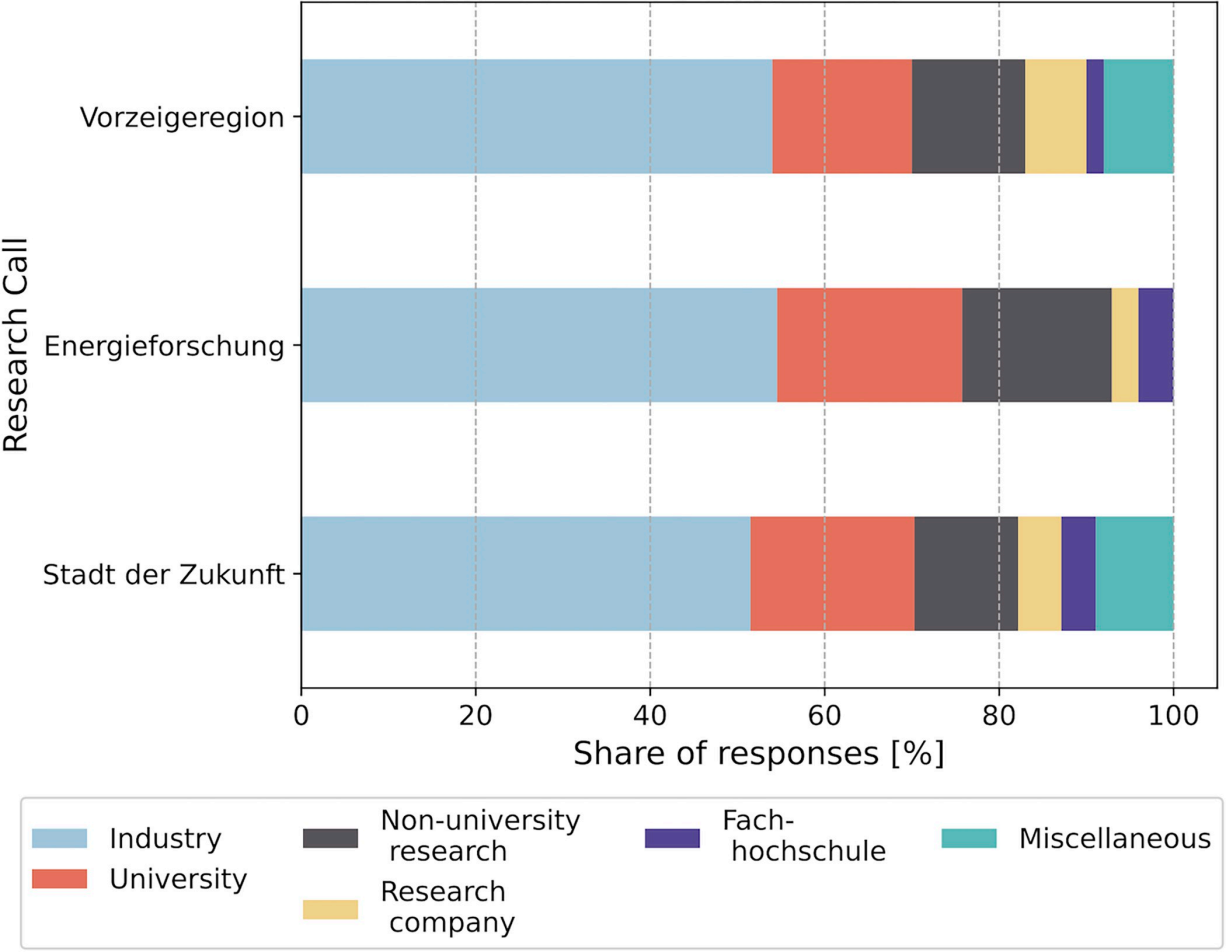
Composition of projects for the research calls Stadt der Zukunft (average for the years 2019, 2020, 2021), Energieforschung (average for the years 2017, 2018, 2019, no data were available for the years 2020 and 2021.), and Vorzeigeregion.

Fig 2 gives an overview of the composition of projects; no distinction is made between project lead and project partners. A total of 158 projects were analyzed. The figure shows a similar distribution for the three research calls. Most of the partners come from industry (more than 50%), followed by partners from the categories "university" (16–21%) and "non-university research" (12–17%).

Table 1 shows the share of project leads per partner category. The table shows a similar distribution for the calls Stadt der Zukunft and Energieforschung, where most projects are led by universities. In call Vorzeigeregion, most projects are led by partners from industry. This could be explained by the fact that projects in the Vorzeigeregion usually have a high Technology Readiness Level (TRL).

**Table 1:** Share of project leads per partner category.

	University	Fachhoch-schule	Non-university research	Research company	Industry	Miscell-aneous
Stadt der Zukunft	31%	10%	31%	5%	20%	3%
Energieforschung	38%	5%	34%	5%	18%	0%
Vorzeigeregion	26%	6%	24%	12%	32%	0%

**Fig 3:**
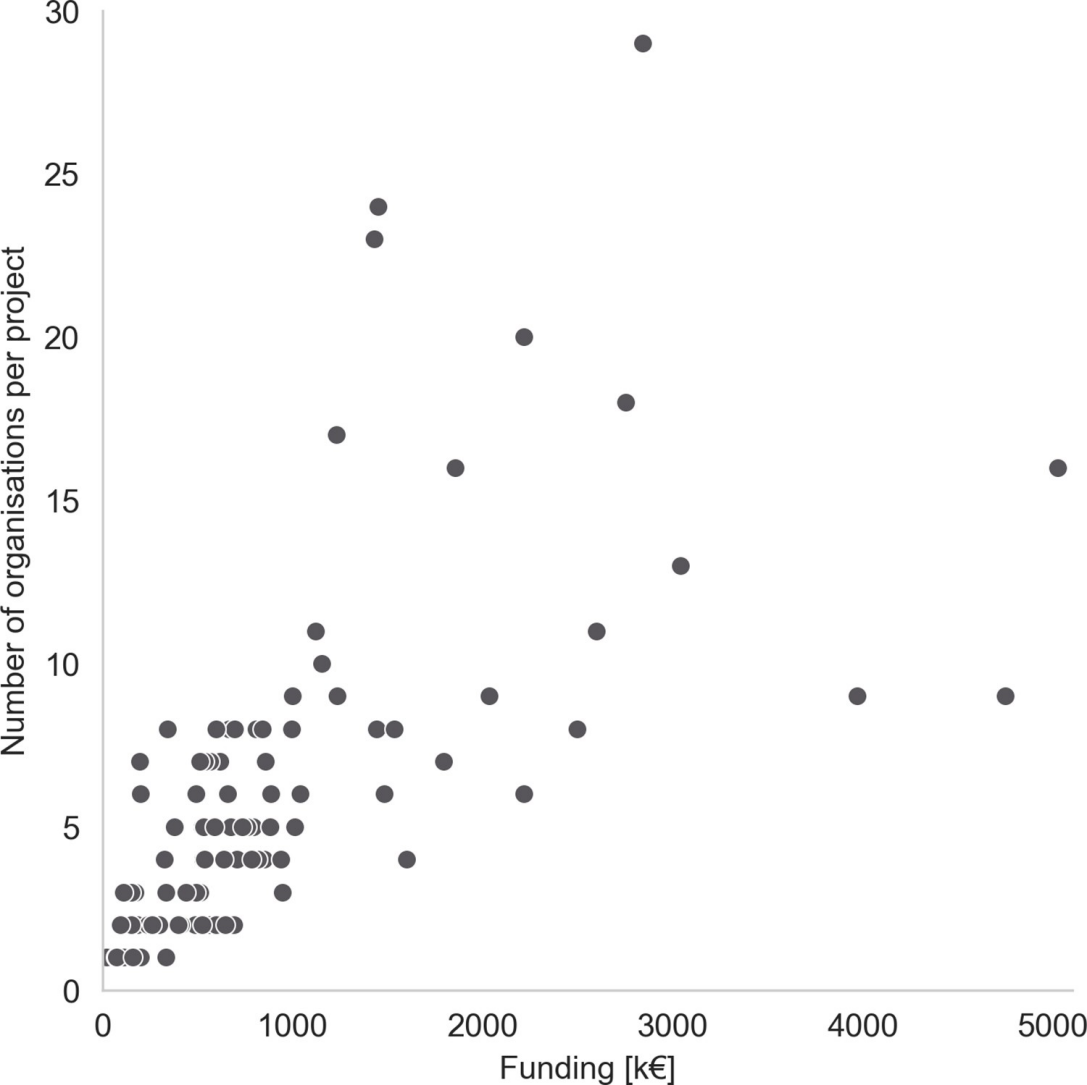
Scatter plot of Number of organizations per project (incl. lead partner) against funding in k€.

Fig 3 gives an overview of the project size in terms of project partners (y axis) and funding volume (x axis). Since no information on budgets was available for the projects in the research call "Stadt der Zukunft", a total of 91 projects from the research calls "Energieforschung" and "Vorzeigeregion" were analyzed. Two projects have a funding less than €100k; 19 projects between €101-300k; 10 projects between €301-500k; 20 projects between €501-700k; 16 projects between €701-1M; 9 projects between €1–1.5M; 3 projects between €1.5-2M; 7 projects between €2-3M, 2 projects between €3-4M, and 1 project each between €4-5M and more than €5M.

After repeated requests to FFG, no information on acceptance rates was disclosed. A report was found that included information on the acceptance rate for the research call “Energieforschung 2018”. In this call, the acceptance rate was about 17% [29].

### 3.2 Empirical results

Of those surveyed in the field of research, 29 responses were from "non-academic research", 18 from "University", 10 from "Fachhochschule", 4 from "research companies" and 2 did not provide any further information. Twenty-four of the respondents were Professor/Chief Investigator/Lab-Head, 19 were post-docs, 19 were researchers without PhDs, and one person did not provide more specific information. Thirty-nine respondents indicated that their most recent FFG project application in the field of energy was submitted to “Stadt der Zukunft”, 32 to “Energieforschung”, 19 to “Vorzeigeregion”, and 13 to "Other."

Of those surveyed in the field of industry, 6 responses were from people working in large companies (over 250 employees), 3 in medium-sized companies (between 50–250 employees), 8 in small-sized companies (less than 50 employees), 2 in start-ups, and 2 in administration. Eight people were in leading positions (CEO, CTO), 6 were in middle management, 5 were working in research and development positions, and 2 did not specify their current position. Six respondents indicated that their most recent FFG project application in the field of energy was submitted to “Vorzeigeregion”, 5 to “Energieforschung”, 4 to “Stadt der Zukunft”, and 6 to "Other”.

#### 3.2.1 Verification

**Fig 4:**
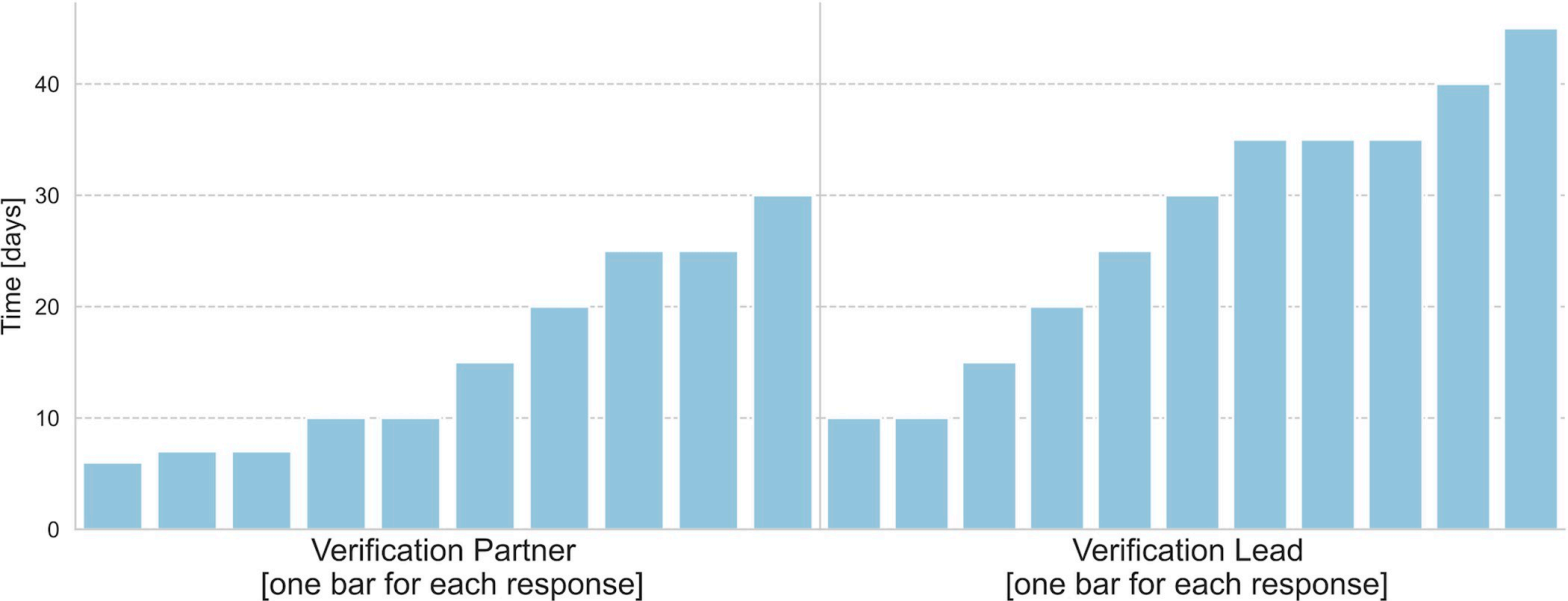
Responses of the verification sample (see chapter 2.2.3) to the question “what total number of days they and their organization in total spent preparing proposals”. Each bar represents one response.

A total of 16 participants responded to the verification. One of 16 respondents mentioned that he/she answered the question in hours rather than days; another mentioned that he/she could not remember exactly whether he/she had given the answer in days or hours. Fig 4 shows the answer of the verification sample to the question “How many days (1 day = 8 hours) did your organization spend, in total (including your time), working on the proposals (as lead and/or partner)?” One respondent answered the question only for “lead”.

#### 3.2.2 Outlier detection and survey data cleaning

*Survey data cleaning*. In total, 125 surveys on projects were received: 50 projects in which research was the lead, 53 projects in which research was partner, and 22 projects in which industry was partner. It should be stressed that respondents from research were able to provide information on projects in which they were lead and/or partner. Therefore, there are more projects than the total number of 85 responses from research and industry.

Seven of 125 responses were inconsistent with respect to the questions “How many days (1 day = 8 hours) did you (just you, not your institution’s team) spend working on the proposal?” and “How many days (1 day = 8 hours) did your organization spend, in total (including your time), working on the proposals?”, because the response to the first question was greater than the second. The author assumes that in these cases, the statement in the bracket "including your days" was overlooked. No responses indicated straightlining. Thus, 118 responses were examined for outliers.

*Outlier detection*. Table 2 shows results of the two outlier detection methods.

**Table 2:** Outlier detection.

	Quartile Q_1_ [days]	Quartile Q_3_ [days]	IQR (= Q_3_- Q_1_) [days]	Mild outlier >*Q*_3_+1.5×*IQR* [days]	Extreme outlier >*Q*_3_+3×*IQR* [days]
Research Partner	10	20	10	> 35	> 50
Research Lead	13	35	22	> 68	> 100

When the "mild outlier" criterion is applied, a total of 13 responses are classified as outliers, and with the "extreme outlier " criterion, 8 responses. The results in Table 3 show that the median for the original data and the median calculated after removing outliers are identical. The results show that the verified respondents have a slightly higher median than the original responses.

**Table 3:** Analysis of the remaining data: Minimum, Maximum, Median, Standard Deviation.

	Minimum [days]	Maximum [days]	Median [days]	Standard Deviation [days]
Research Partner “Original Data”	1	200	12	42
Research Partner “Extreme outlier”	1	48	12	10
Research Partner “Mild outlier”	1	30	12	8
Research Lead “Orignial Data”	2	300	20	61
Research Lead “Exteme outlier”	2	100	20	19
Research Lead “Mild outlier”	2	45	20	10
Verification Research Partner	6	30	12.5	9
Verification Research Lead	10	45	30	12

**Fig 5:**
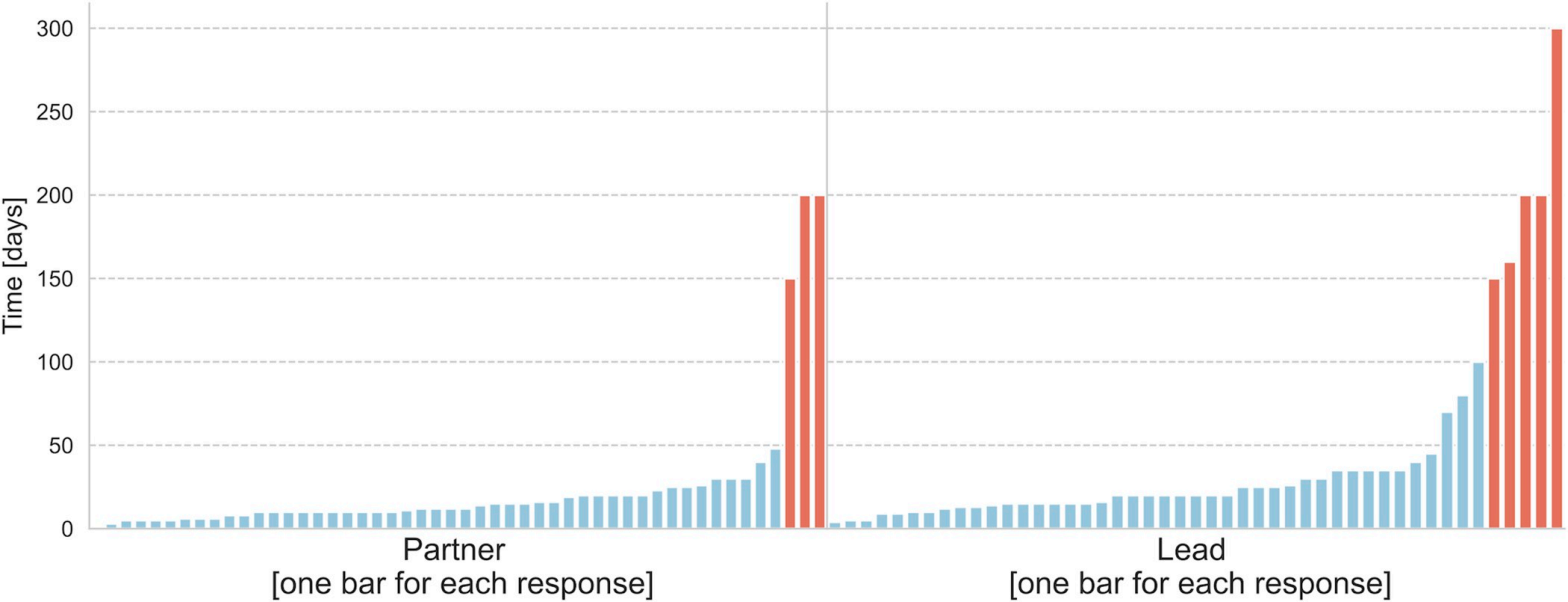
Responses to the question “How much time in days did your organization spend working on the proposal overall (including your days)?”.

In the further course of this work, the criterion “extreme outlier” is applied for the detection of outliers. Fig 5 shows the original responses to the question, “How many days (1 day = 8 hours) did your organization spend, in total (including your time), working on the proposals?” Responses greater than 50 for project partner and greater than 100 for the lead were defined as outliers. Outliers were removed for the analysis in the following chapters. In these cases, however, not all answers of the respective person were removed, but only the answers to time-related questions.

#### 3.2.3 Time expenditure per project

**Fig 6:**
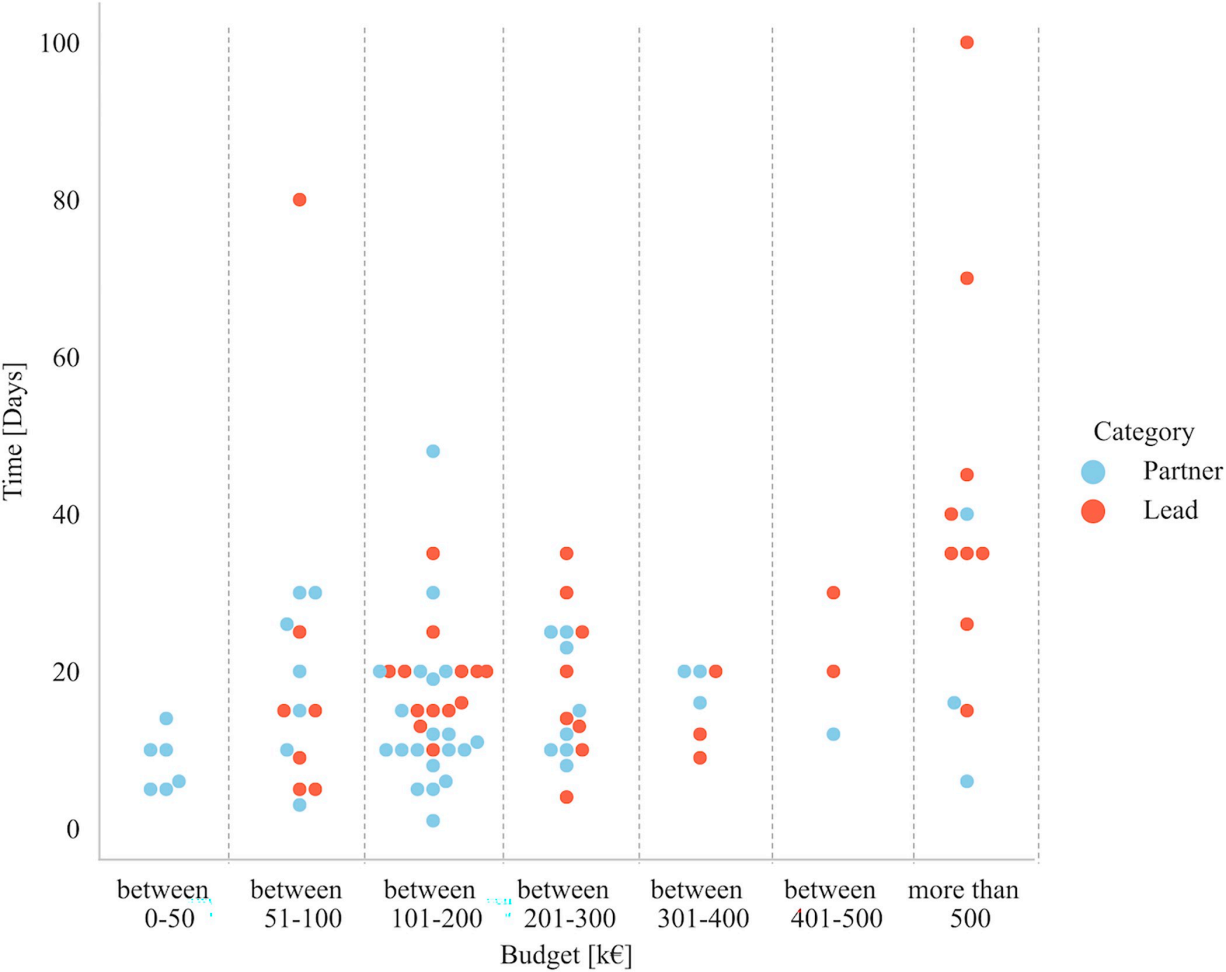
Days (as a continuous variable) spent by the researchers’ organization to prepare a research proposal either as partner (blue) or as lead (red) for the corresponding budget (as a categorical variable).

Participants from research were asked to provide an estimate of the total number of days they and their organization spent preparing proposals; separately, as project lead and as a project partner. Industry participants were asked the same questions, but only for their role as a project partner. Additionally, all respondents were asked if it was a new proposal or a resubmitted proposal. Days (continuous variable) spent by the researcher’s organization to prepare a research proposal either as partner (blue) or as lead (red) for the corresponding budget (as a categorical variable) is presented in Fig 6.

**Fig 7:**
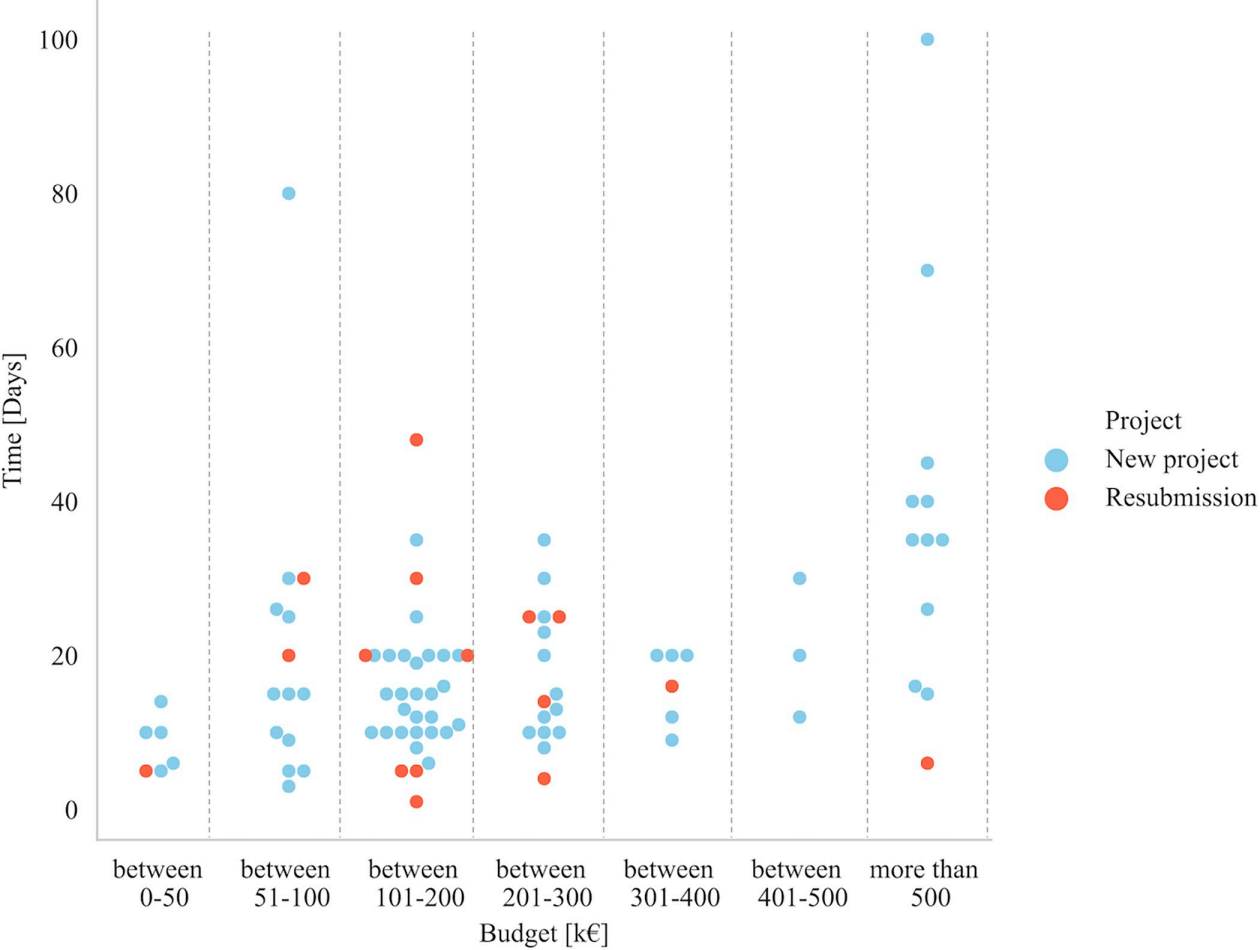
Days (as a continuous variable) spent by the researchers’ organization to prepare either a new (blue) or resubmitted (red) research proposal for the corresponding budget (as a categorical variable).

Days (continuous variable) spent by the researchers’ organization to prepare either a new (blue) or resubmitted (red) research proposal for the corresponding budget (as a categorical variable) is presented in Fig 7. Results indicate that no correlation can be made between these variables, but more data are needed to confirm this observation.

Table 4 shows results regarding the time required for preparing of new projects compared to resubmissions for different categories. The mean and median are close to each other; this shows that the data are symmetrically distributed. For the categories "Research–Lead—Resubmission" and "Industry–Partner—Resubmission" there are too few responses to derive general conclusions. Based on the results for "Research–Partner—New Submission" and "Research–Partner—Resubmission," it is assumed in the following that there are no differences for the two categories. However, future research needs to confirm this assumption (see also Section 2.2.4).

**Table 4:** Responses to the question “How much time in days did your organization spend working on the proposal overall (including your days)?” for different categories.

	Median [days]	Mean [days]	Number of responses
Research—Lead—New submission	20.0	24.2	41
Research—Lead—Resubmission	17.0	15.8	4
Research—Partner—New submission	11.5	13.7	38
Research—Partner—Resubmission	18.0	17.6	12
Industry—Partner—New submission	5.5	6.3	18
Industry—Partner—Resubmission	16.5	15.8	4

The three research calls analyzed in the paper offer (like most other FFG research calls) three options for submitting projects (referred to as “project category”) that follow the technology readiness concept [30]. (a) Industrial research (TRL 2–4), (b) experimental development (TRL 5–8) and (c) exploratory studies, which serve to prepare research, development and innovation projects. Funding rates as a percentage of project costs range from 35–60% for projects in the experimental development category, 50–80% for exploratory studies, and 55–85% for projects in the industrial research category. Research institutions receive the highest funding rate, while large companies receive the lowest. [31]. A total of 116 responses were given in the survey regarding the project category. In 51% of the cases the project was submitted in the category "industrial research", in 31% of the cases "experimental development", in 15% of the cases "exploratory", and 3% "other". The time it takes researchers to prepare proposals for the different categories is similar. Results of the survey show that “research” as project partners need a median of 10 days for preparing proposals in the category experimental development, 16 days for projects in the categories industrial development, and 15 days for projects in the category exploratory studies. These results are not surprising, as the application documents for the three project categories are nearly identical (see e.g. [32]). This is especially relevant since exploratory projects are mostly limited with a maximum funding budget of €200.000 per project (see. e.g. [33]).

#### 3.2.4 On the time spent preparing a hypothetical proposal

In the following, an analysis is given of how much time would have to be spent on a hypothetical project, based on the findings of the previous chapters. According to the survey results, the average number of project partners (including the lead partner) is 5.11; this is in line with the results of the analysis of the calls (data based on research project databases; see chapter 2.1), where the average is 5.14. Based on data from the project databases, the average projects consists of 0.88 partners from "university", 0.18 from " Fachhochschule", 0.75 from "non-academic research", 0.26 from a "research-oriented company", 2.78 from "industry", and 0.28 from “miscellaneous”. For the analysis in the following, "University", "Fachhochschule", "non-academic research", and "research oriented company" are grouped under the term "research", and "industry" and "miscellaneous" are grouped under the term "industry". Thus, the hypothetical project team consists of 2.1 partners from “research” and 3.1 partners from “industry”. It is assumed that the hypothetical project is led by a partner from research (in line with the results in Table 1). The assumption regarding the time needed to prepare the proposal is based on the median values (according to the results in Table 3, no distinction is made between new projects and resubmissions): 20 days for “Research-Lead”, 12 days for “Research-Partner”, and 6 days for “Industry-Partner”. Based on these assumptions, a total of 52 days are needed to prepare a hypothetical proposal. Table 5 shows median, means, and confidence intervals for the aggregated measures of time estimations.

**Table 5:** Aggregated data: Medians, Means and 95% confidence intervals.

	Median [days]	Mean [days]	CI upper (95%) [days]	CI lower (95%) [days]
Research—Lead	20.0	24.8	30.8	18.7
Research—Partner	12	15.0	17.8	12.1
Industry—Partner	6.0	8.4	12.1	4.7

**Fig 8:**
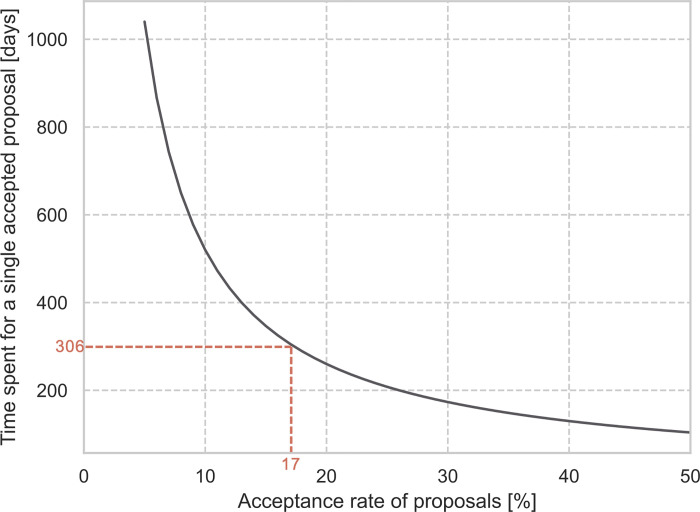
The time required to write hypothetical proposals as a function of the acceptance rate in order to fund a single hypothetical project. The acceptance rate of 17% is the acceptance rate of the research call Energieforschung in 2018 [29].

Fig 8 shows the time required to write hypothetical proposals as a function of the acceptance rate, in order to fund a single hypothetical project; for example, with an acceptance rate of 25%, 4 projects (corresponding to 208 days of work) must be submitted for one to be funded. The author assumes that this is a conservative estimate, as it can be assumed that for every research call there will be projects that are written up to a certain stage, but then not submitted.

#### 3.2.5 Research proposals per year

**Fig 9:**
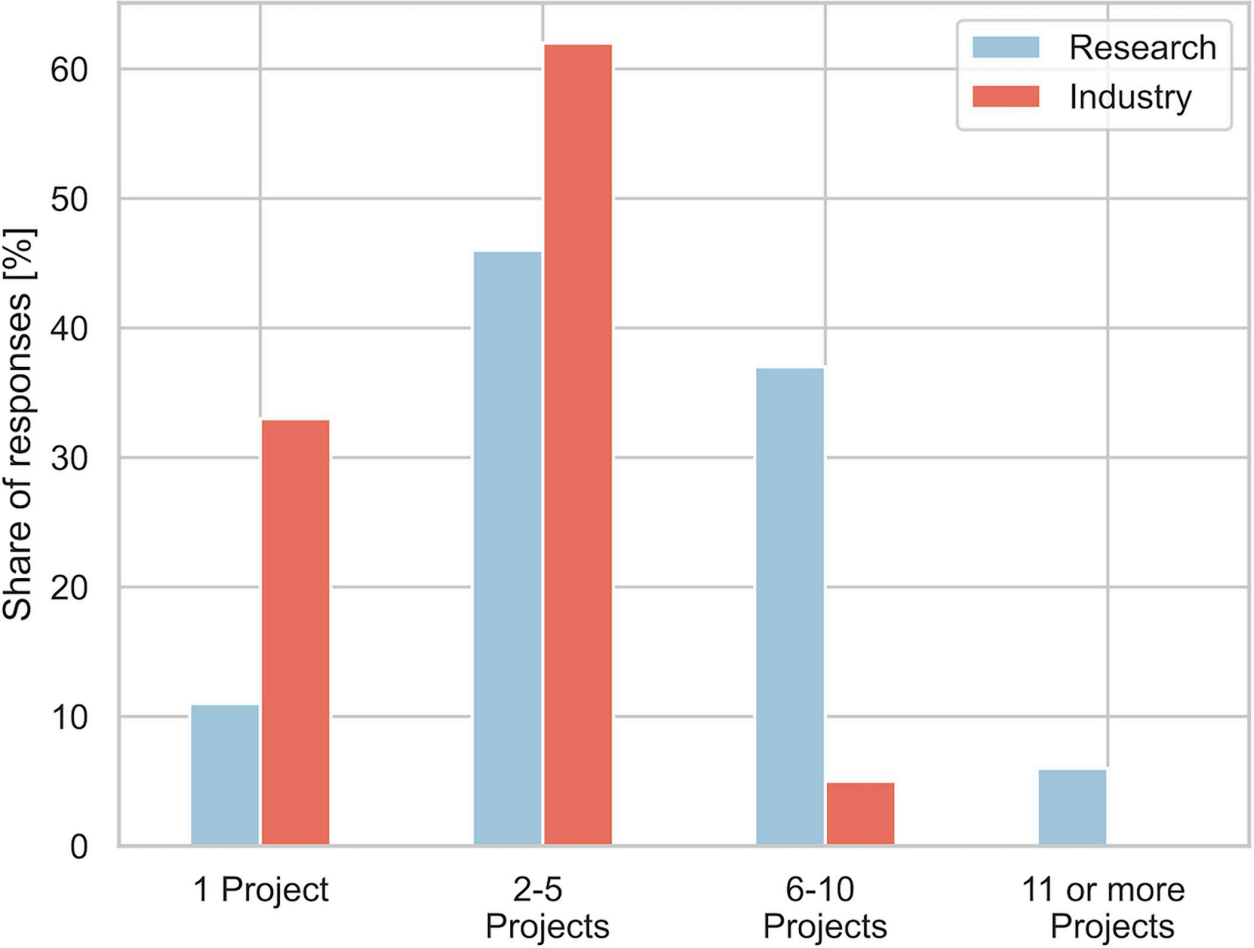
Share of responses for the different categories; responses were collected as single choice for the categories shown on the x axis. Each bar represents the share of responses for the respective category.

Respondents were asked, "How many research proposals have you personally been involved in over the past 12 months?"; the results are shown in Fig 9.

Over 40% of researchers work on more than 6 applications per year. The median time it takes researchers to prepare proposals (regardless of lead or partner) is 13 days. With 6 applications, this totals 78 days per year, which corresponds to 31% of working days—which are, on average, 251 per year in Austria; 10 applications correspond to 52% of the time.

#### 3.2.6 Likert-scale questions

**Fig 10:**
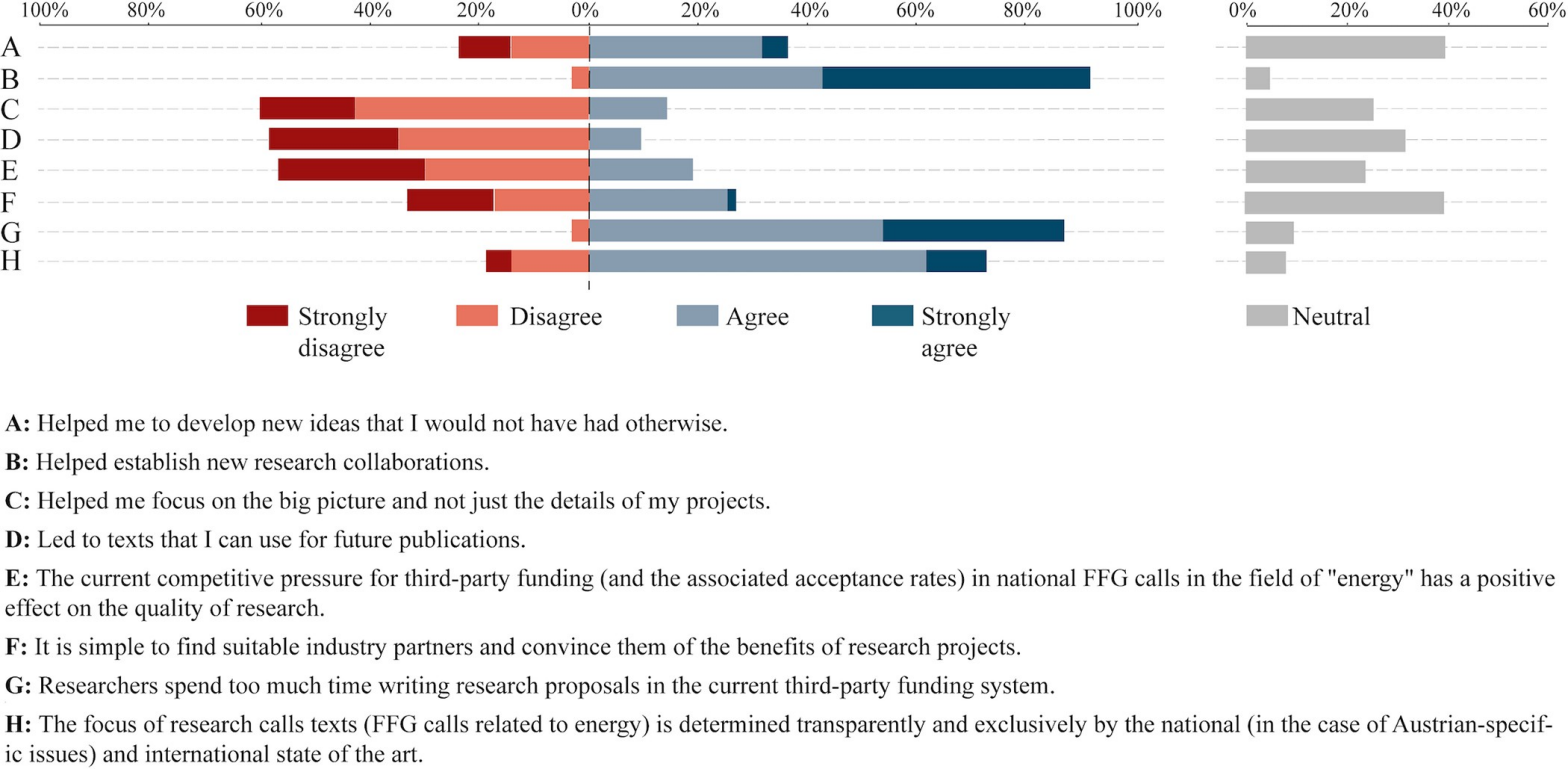
Responses from research based on a 5-point Likert scale.

**Fig 11:**
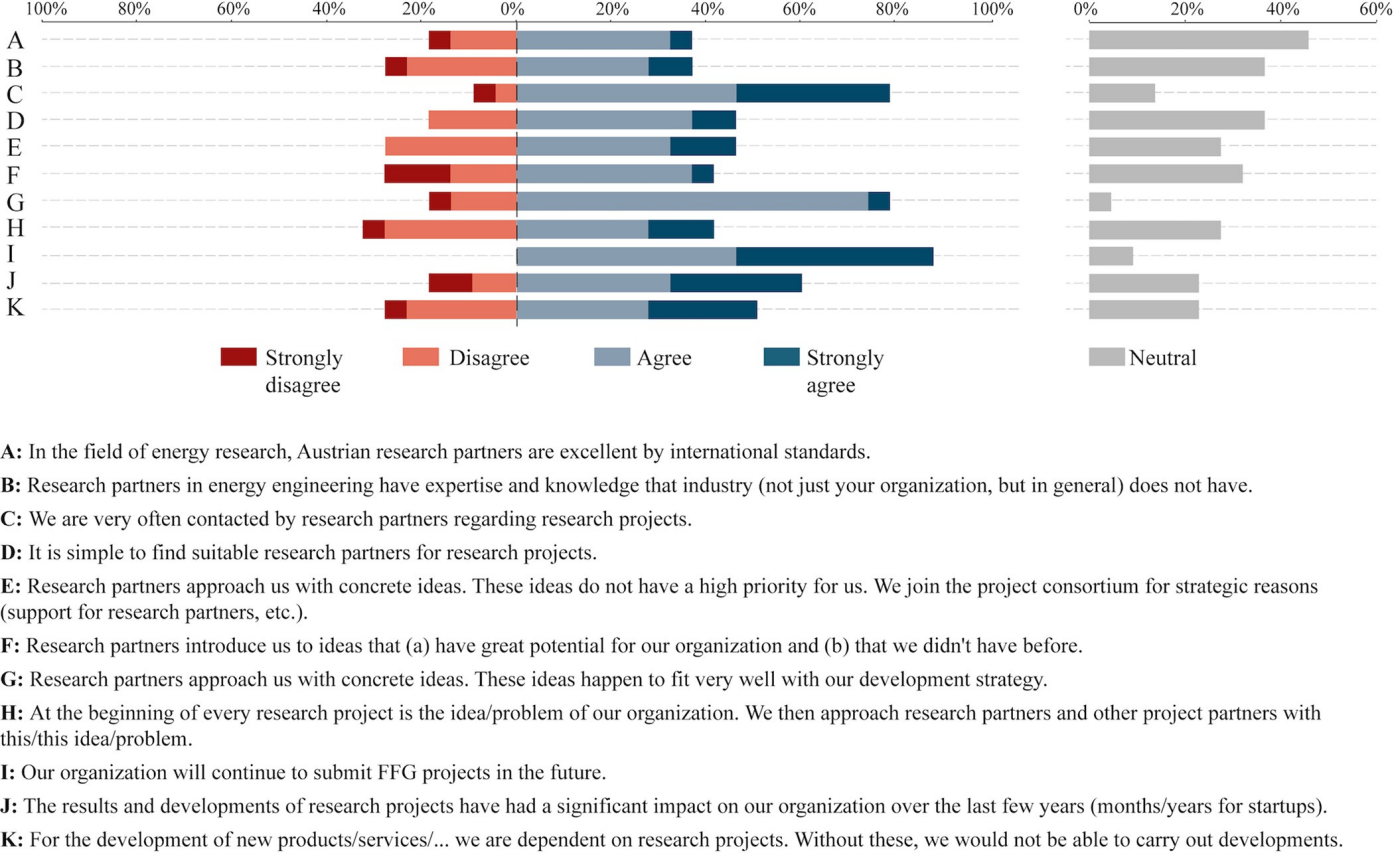
Responses from industry based on a 5-point Likert scale.

Results from the 5-point Likert-scale questions are presented using diverging stacked bar charts in Fig 10 (for research) and Fig 11 (for industry). Since the neutral responses are visualized separately, it is easier to identify trends regarding agreement and disagreement with certain statements. Respondents were asked to indicate how much they agreed with certain statements.

Ninety-two percent of researchers strongly agree or agree that a researchers spend too much time writing proposals in the current third-party funding system, and this even though proposal writing has significant benefits that researchers acknowledge, including agreement that writing proposals helps them to develop new ideas or that it leads to new collaborations. This is in line with findings from other studies (see e.g. [16]). Ten percent of researchers agree or strongly agree with the statement that “the current competitive third-party funding systems has a positive effect on the quality of research”. Fourteen percent of researchers agree or strongly agree with the statement that “it is simple to find suitable industry partners and convince them of the benefits of research projects”. This is in line with the fact that 77% of the respondents from industry agree or strongly agree with the statement that “we are very often contacted by research partners regarding research projects”. Forty-six percent of respondents from industry agree or strongly agree that they join projects for strategic reasons (e.g. to support research partners) and not because projects are important for them; on the other hand, 41% of industry respondents agree or strongly agree that research partners introduce new ideas that have great potential for them. Sixty percent of respondents from industry agree or strongly agree with the statement that research projects have had a significant impact on their organization in the past few years. Thirty-six percent of respondents from industry agree with the statement that research partners have expertise and knowledge that industry is lacking; likewise, 36% agree or strongly agree that Austria’s research partners are excellent by international standards.

#### 3.2.7 Reliability of peer-review

**Fig 12:**
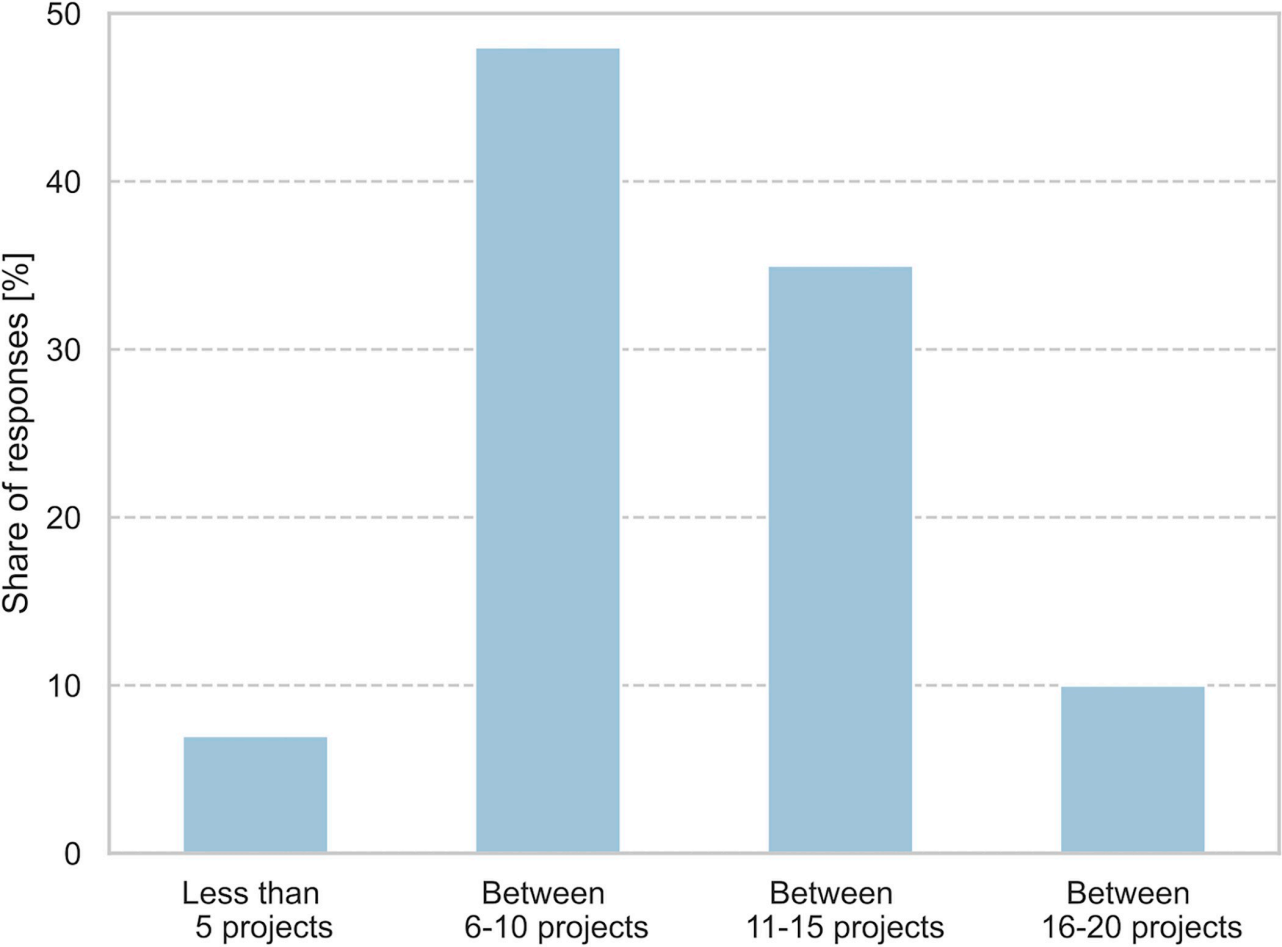
Share of responses for the different categories; responses were collected as single choice for the categories shown on the x axis. Each bar represents the share of responses for the respective category.

Researchers were asked to imagine a scenario in which 100 project proposals in their research field have been reviewed by a "Panel A" of 10 experts. Panel A has selected 20 applications for funding. In a second step, respondents should then imagine that a second panel of 10 experts (Panel B; consists of different experts as Panel A) reviews the same 100 projects and must independently decide which 20 proposals should receive funding. Respondents were then asked how many of the 20 proposals originally selected by “Panel A” they would expect also to be selected by “Panel B” (the question was adopted from [16]); Fig 12 shows the responses. More than 50% of researchers see a 50% or more disagreement in funding between two hypothetical review panels. The results differ from the findings of Herbert et al. [16] (whose answers were not given in categories, but in a number between 0–20) whose respondents most frequently selected 15 out of 20, or a 25% disagreement.

FFG uses different evaluation procedures in its selection process [34]. In the case of competitive funding, a central element is a so-called evaluation committee, which is composed of national and/or international experts. In addition, the FFG can request complementary peer reviews if necessary, which are also made available to the members of the evaluation committee. An additional review stage was introduced for submissions to the "Vorzeigeregion”. As a first step, there is an internal pre-selection by a so-called steering group, which consists of 19 members from research and industry [35]. These partners are all eligible to submit their own projects, but cannot evaluate them in the first stage [36]. It is questionable whether this process will increase trust in the objectivity of the review process, especially among organizations that are not among the 19 members of the steering group.

## 4 Discussion

An objective and comprehensive comparison between competing research funding seems to be difficult; according to the author, it is scientifically dishonest to summarize the results in a sweeping but empirically untenable "better" or "worse." A candidate metric for comparing different systems would be citation counts, for which a number of evaluation metrics have been proposed [37–39]. Citation count analysis, however, assumes that each citation in a paper is to be considered equal in terms of the contribution it has to the citing paper [40]. In reality, this is not the case, as citations can be diverse concerning their function or polarity. A companion to purely quantitative measures is so-called citation context analysis, which aims to obtain better understanding of the link between citing and cited work [41]. Citation context analyses qualitatively interpret the symbolic information embedded in the text surrounding citations [42]. Al-Ashaab et al. propose a scorecard to capture the impact of collaborative research projects for industries [43]. It has different performance indicators in six perspectives: competitiveness, sustainable development, innovation, strategic knowledge partnership, human capital and internal business processes. Rezapour et al. propose a method for evaluating the impact of funded research beyond academia that takes into account social and economic aspects [44]. Schubert defined 12 measures that determine a research output: publications, citations, conference articles, international co-publications, professorial job offers, advisory services for companies, cooperation with companies, membership in advisory boards, number of doctoral titles, number of state doctoral theses, editorships, and scholarships [45].

There are many studies on the impact of competitive research funding on productivity related to publications (see [46] for an overview). However, the external validity of these studies depends on the similarity of the funding system, [47] and results from Auranena and Nieminen show that there are significant country-specific differences among university systems in relation to steering impulses and competition incentives [4]. The author is not aware of any study where this analysis was explicitly conducted for the Austrian system. However, Bolli and Somogyi explicitly mention that the Swiss system they study is very similar to the Austrian system [47]. They analyzed the impact of private and public third-party funding on the productivity of Swiss research. They found no impact of third-party funding on teaching productivity. The share of third-party funding improves publication productivity. Technology transfer productivity is independent of public third-party funding, but is increased by private funding.

Auranena and Nieminen examined how different funding systems vary across countries and whether more competitive funding systems have a positive impact on productivity in terms of scientific publications [4]. They conclude,“*The efficiency calculations suggest*, *in turn*, *that the idea of output and competition-based incentives promoting productivity in science is more complex than policy-makers seem to believe*. *Even though the countries with a competitive funding environment for university research (the UK*, *Australia and Finland) appear more efficient than the rest*, *they have not been able to increase their efficiency in publication output*. *At the same time*, *some university systems with a less competitive funding environment are either almost as efficient as the more competitive systems (Denmark) or have been able to increase their efficiency despite the relatively low level of competition for funding (Sweden and Germany)”*. Furthermore, they argue that too much competition can have a negative impact on productivity, as competition for grants takes an excessive amount of time and energy away from research and writing. They also ask the question of whether incentives that have traditionally been an integral part of science (e.g., researcher reputation) are more important than funding-related incentives.

Sandström and Van den Besselaar analysed how competitive funding effects efficiency [6]. They defined efficiency as the change in funding compared to the change of top-cited papers within a particular research field and competitiveness as the share of competitive project funding within the total funding. They then analyzed the relationship between those two measures over ten years among 17 countries. The results show a small negative correlation. The authors state that given the small amount of data, the results should be used with extreme care.

It is important to emphasize at this point that competitive research has an impact that goes far beyond mere citation. Boudreau et al. found that peer review evaluators give lower scores to research proposals that are closer to their own research and that more novel proposals are associated with lower evaluations [48]. This leads to a situation in which too much of funding goes to research projects with predictable results instead of highly innovative projects. Herbert et al. analyzed the impact of a competitive funding environment on researchers’ productivity, health and well-being [49]. They conclude that writing grants for a single annual deadline is stressful, time-consuming, and conflicts with family responsibilities. However, it is not clear whether the evaluation of researchers is similar in a competitive system with multiple deadlines per year; this should be investigated in future research.

## 5 Conclusion

This article presents an analysis of third-party funding in Austria in the field of energy research. This includes an analysis of survey responses regarding the effect of the current third-party funding system on proposal applicants and their trust in the review process. On average, a project consists of about 5 different partners from research and industry working together for 52 days on a single proposal. With a 17% acceptance rate, about 300 working days (1.2 years) must be spent on preparing proposals in order for a single project to be funded. Ninety-two percent of researchers believe that they currently spend too much time preparing proposals and only 10% of researchers believe that the current competitive third-party funding systems have a positive effect on the quality of research. The potential for a more efficient funding system is immense; if researchers could spend more time on actual research, it would be more likely that there would be more developments and innovations in less time.

Grant review procedures have great influence on the direction of research and on the research path of many researchers. On the one hand, empirical studies have shown that chance has a significant impact on funding decisions [7–10]. On the other hand, studies such as the present one show that researchers do not trust in the peer review process. More than 40 years after Cole and colleagues questioned whether a system in which funding decisions depend to a significant degree on chance is the most rational, many funding agencies seem to be doing little to update themselves despite compelling empirical evidence. Due to the statistical significance of chance in funding, it is still rational for researchers to submit as many applications as possible, as this increases the likelihood of receiving funding.

Recommendations:

- Simplify project submission processes, e.g., by establishing staged application processes [16].
- Consider different distribution strategies such as lottery-based systems [50].
- Increase base funding of universities and decrease competitive third-party funding [51,52].

Loosely speaking, we can conclude that we as researchers spend too much time on tedious work, whose outcome depends very much on chance, which negatively impacts the quality of research. Many alternative approaches have been proposed that would potentially improve the situation, many of which have not even been considered and/or implemented. The government and its funding agencies would do well to heed the research and implement its findings.

Future work should investigate the relation between direct investments in green technologies and sustainable systems, and applied research. The author believes that in order to transform the energy system in the direction of a smart, sustainable system, we need (a) funding for basic research to stimulate radical innovations and (b) direct investment into innovative systems and technologies.

It needs to be clarified whether it is only good for quotas and statistics, or whether it is a good prerequisite for conducting high-excellence research at an international level if a large portion of research funding is allocated to applied research, in which a large share is spent on direct investments.
